# An Unusual Case of Aortic Vegetation Causing Coronary Artery Microembolization and Sudden Death: A Case Report

**DOI:** 10.7759/cureus.83260

**Published:** 2025-04-30

**Authors:** Mohamed R Mohamed, Hayley Mitchel, Farouk Mookadam, Radha Gopalan

**Affiliations:** 1 Cardiology, University of Arizona College of Medicine - Phoenix, Phoenix, USA; 2 Radiology, University of Arizona College of Medicine – Tucson, Tucson, USA

**Keywords:** aortic vegetation, cad, endocarditis, iv drug-use, sudden cardiac death (scd)

## Abstract

Infective endocarditis (IE) is a life-threatening condition with increasing prevalence and high mortality, particularly among intravenous drug users (IVDU). While heart failure is a common complication, sudden cardiac death (SCD) due to coronary embolization is a rare event. We report a case of a 35-year-old male with a history of IVDU who presented with fever, chest discomfort, and dyspnea following a traumatic burn injury. Blood cultures grew methicillin-sensitive *Staphylococcus aureus*, and transthoracic echocardiography (TTE) revealed severe aortic valve endocarditis with large vegetations. The patient developed acute coronary occlusion involving multiple branches, with echocardiographic imaging confirming a vegetation prolapsing into the left main coronary artery. Despite appropriate medical management of the IE, he suffered cardiac arrest prior to being able to undergo surgery, presumed to be due to the recurrent acute coronary event related to vegetation obstruction or embolization. Coronary embolization from IE remains an underrecognized but severe complication, with limited reported cases of SCD due to vegetation prolapse into the coronary arteries. This case highlights the importance of early recognition of embolic complications in IE, especially in high-risk patients such as those with IVDU and large vegetations with embolic risk, and underscores the potential need for urgent surgical intervention to prevent fatal outcomes.

## Introduction

Endocarditis has been increasing in prevalence over the past 30 years, from 478,000 cases in 1990 to 1,090,530 cases in 2019. Mortality has also increased for this disease, up to 25% ​[1]​. The diagnosis of infective endocarditis (IE) is made using the Duke criteria with a sensitivity of 83% and specificity of 71% ​[2]​. Empiric treatment is initiated immediately in symptomatic patients with a presumptive positive or definite diagnosis of IE, as the hospital mortality is 20%, and five-year mortality may be as high as 40% ​[3]​. IE can lead to multi-organ complications, including cardiac, neurological, musculoskeletal, renal, and pulmonary complications ​[4-6]​. Heart failure can result from infection-induced valvular damage and, rarely, from vegetation embolization obstructing the coronary arteries, resulting in cardiac ischemia and sudden cardiac death (SCD) from various mechanisms, including coronary embolism, arrhythmia secondary to myocardial damage, severe valvular dysfunction, or myocardial abscess formation. Diagnosing coronary embolism in IE is challenging due to the rarity of the complication, the difficulty in differentiating it from other causes of acute coronary syndrome, and the need for a high index of suspicion, requiring a multidisciplinary approach and advanced imaging [7]. In a recent study by Cooper et al., IE accounted for only 0.5% of 6,000 SCD cases. Pathological examination of this cohort identified four major cardiac causes of mortality related to IE: aneurysm formation, septic emboli leading to myocardial infarction, damage to the membranous septum, and complications from healed endocarditis ​[8]. Here, we present a rare and unusual case of vegetation on the aortic valve that protruded into the left main coronary artery, resulting in cardiac arrest.

"This article was previously posted to the Authorea preprint server on October 19, 2024."

## Case presentation

**Figure 1:**
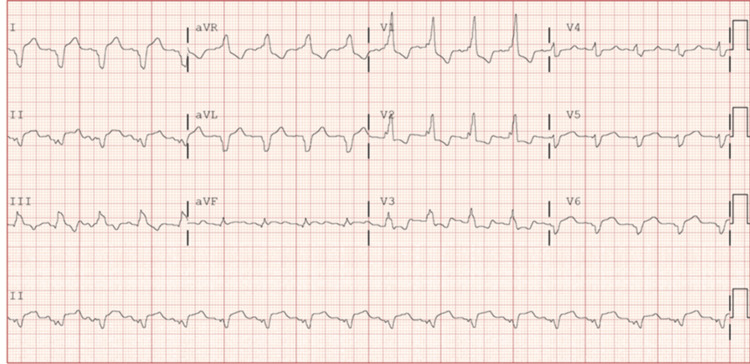
RBBB and ST elevation in lead I, aVL, V5, ST depression in V2 and V3 RBBB: right bundle branch block; aVL: augmented vector left

**Figure 2:**
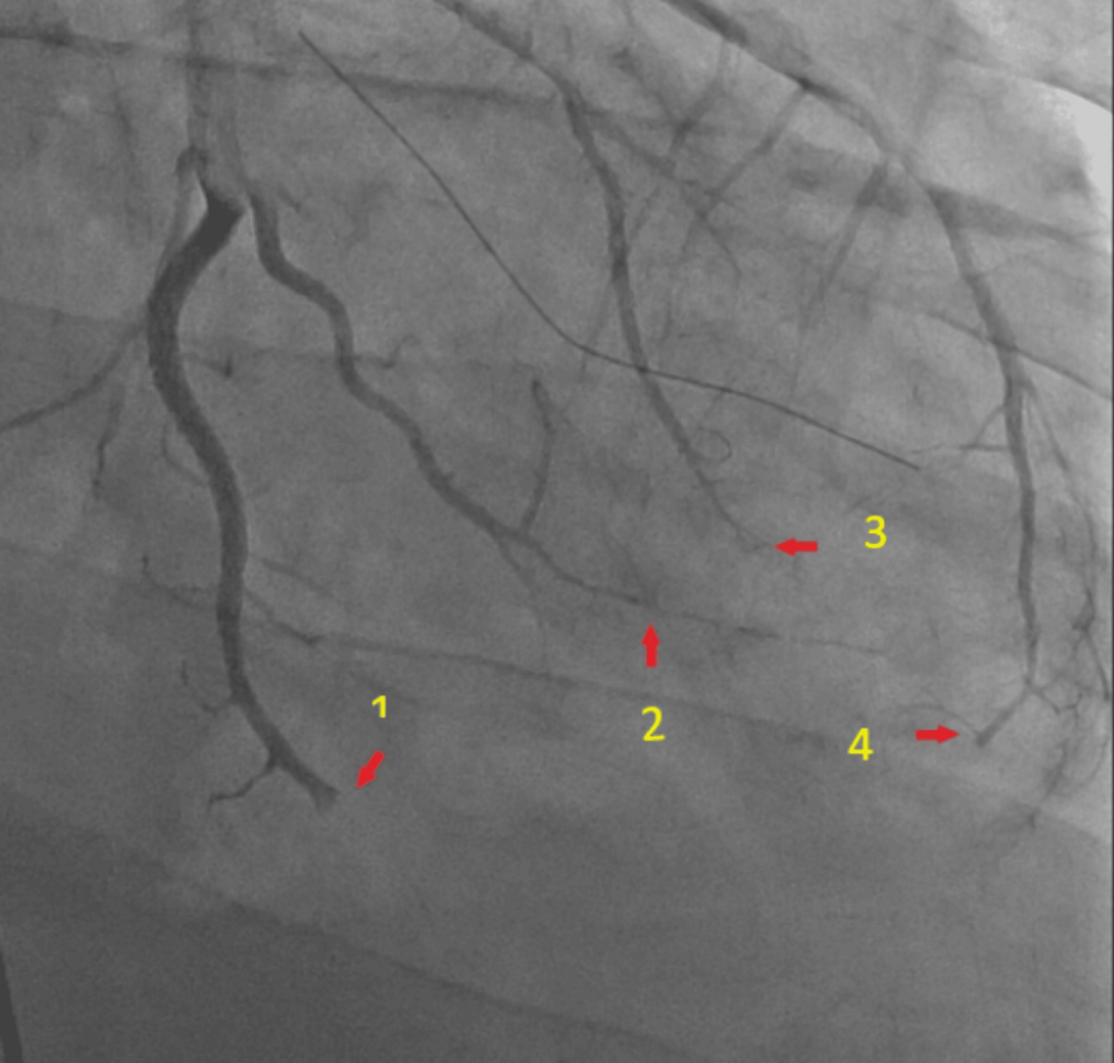
Abrupt truncation of LAD (1) and circumflex system (2,3,4) secondary to embolization from vegetation LAD: left anterior descending

A 35-year-old male with a history of intravenous drug use (IVDU) presented to the emergency room in an outlying facility with chest discomfort, shortness of breath, and generalized weakness after sustaining a burn on his right lower extremity after a motorcycle accident. Upon admission, the patient was febrile with a deep right lower extremity wound. A preliminary drug screen was positive for opioids, and the patient admitted to current intravenous heroin use. Blood white cell count was elevated with neutrophilia, and blood cultures were positive for methicillin-sensitive *Staphylococcus aureus* (MSSA) on two separate occasions, with the presumed source being the lower extremity injury. The patient was placed on appropriate antibiotics. Subsequently, the patient developed chest pain and dyspnea. An electrocardiogram showed a right bundle branch block (Figure 1) and a marked elevation in the high-sensitivity troponin to 6200 (normal <14 ng/L), raising concern for acute coronary syndrome. The patient underwent emergent left heart catheterization that showed abrupt thrombotic occlusion involving the distal circumflex, the obtuse marginal branch of the circumflex coronary artery, an apical left anterior descending (LAD) occlusion, and small diagonal branch occlusion (Figure 2). These findings suggested a widespread thrombotic process involving multiple distal coronary territories, consistent with an embolic phenomenon. A transthoracic echocardiography (TTE) revealed a left ventricular ejection fraction (LVEF) of 20-25% with regional wall motion abnormalities and incidental right ventricular systolic dysfunction. The patient was transferred to our facility for a higher level of care.

**Figure 3:**
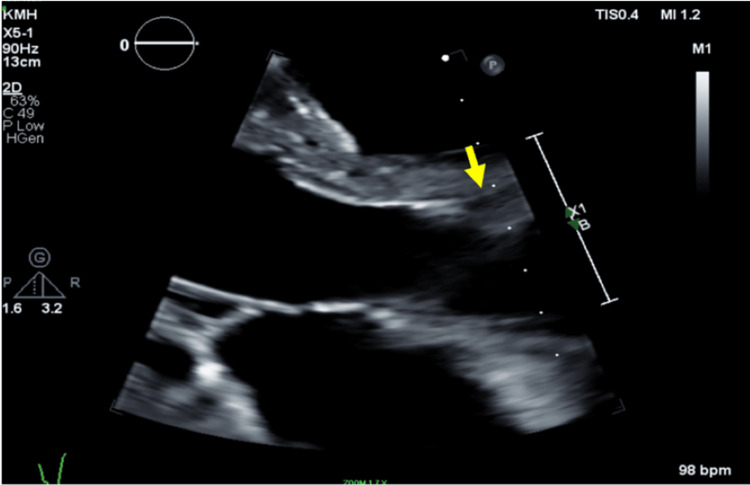
Transthoracic echocardiography (TTE) findings TTE showing parasternal long axis view of the aortic valve and proximal aorta, leaflets are open during systole. Arrow pointing to the right coronary ostium.

**Figure 4:**
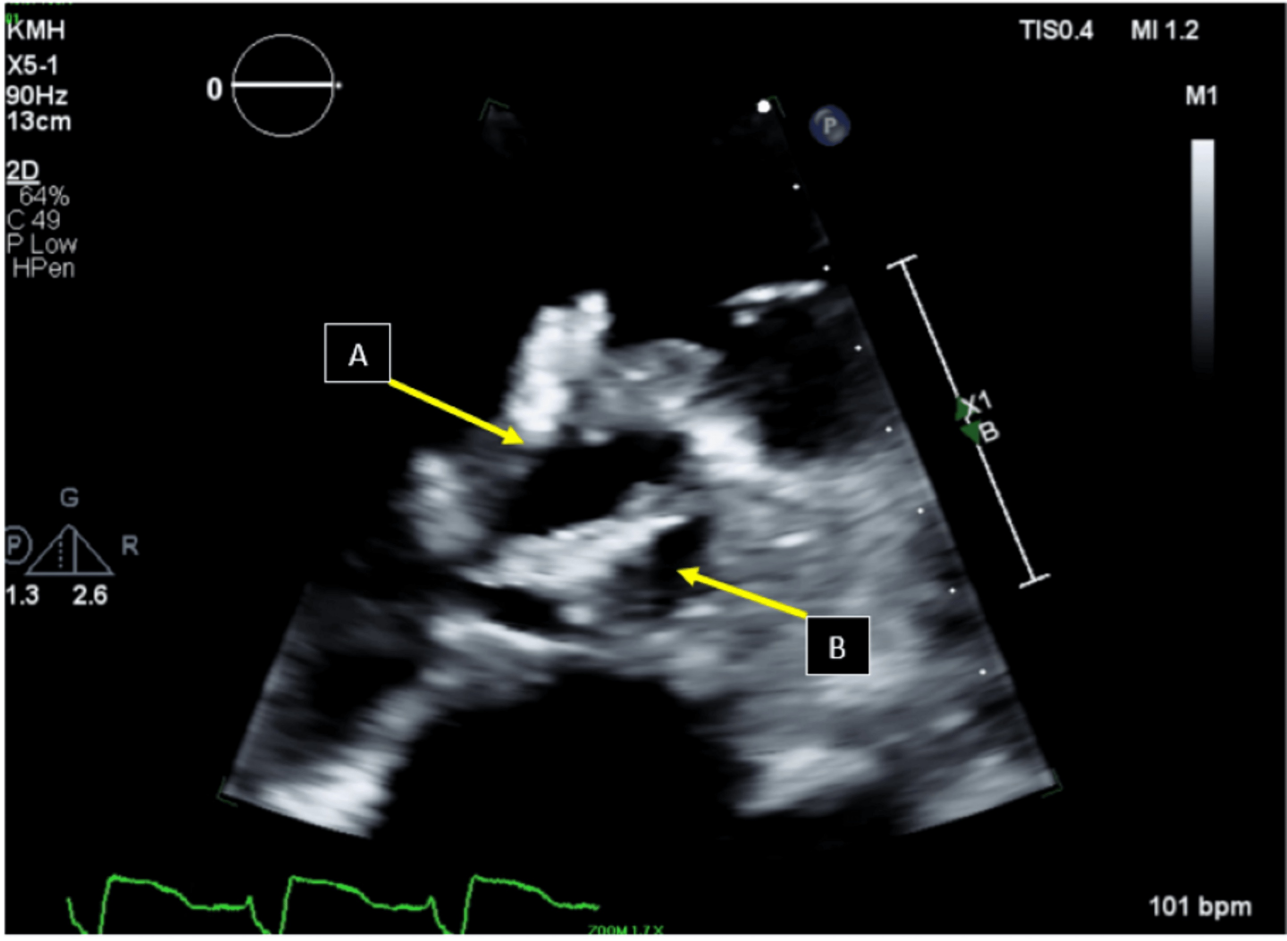
Transthoracic echocardiography (TTE) findings TTE showing a short-axis view of the aortic valve, showing large vegetations on the right coronary cusp (arrow A: RCC). Arrow B shows a large vegetation on the non-coronary cusp (arrow B: NCC).

**Figure 5:**
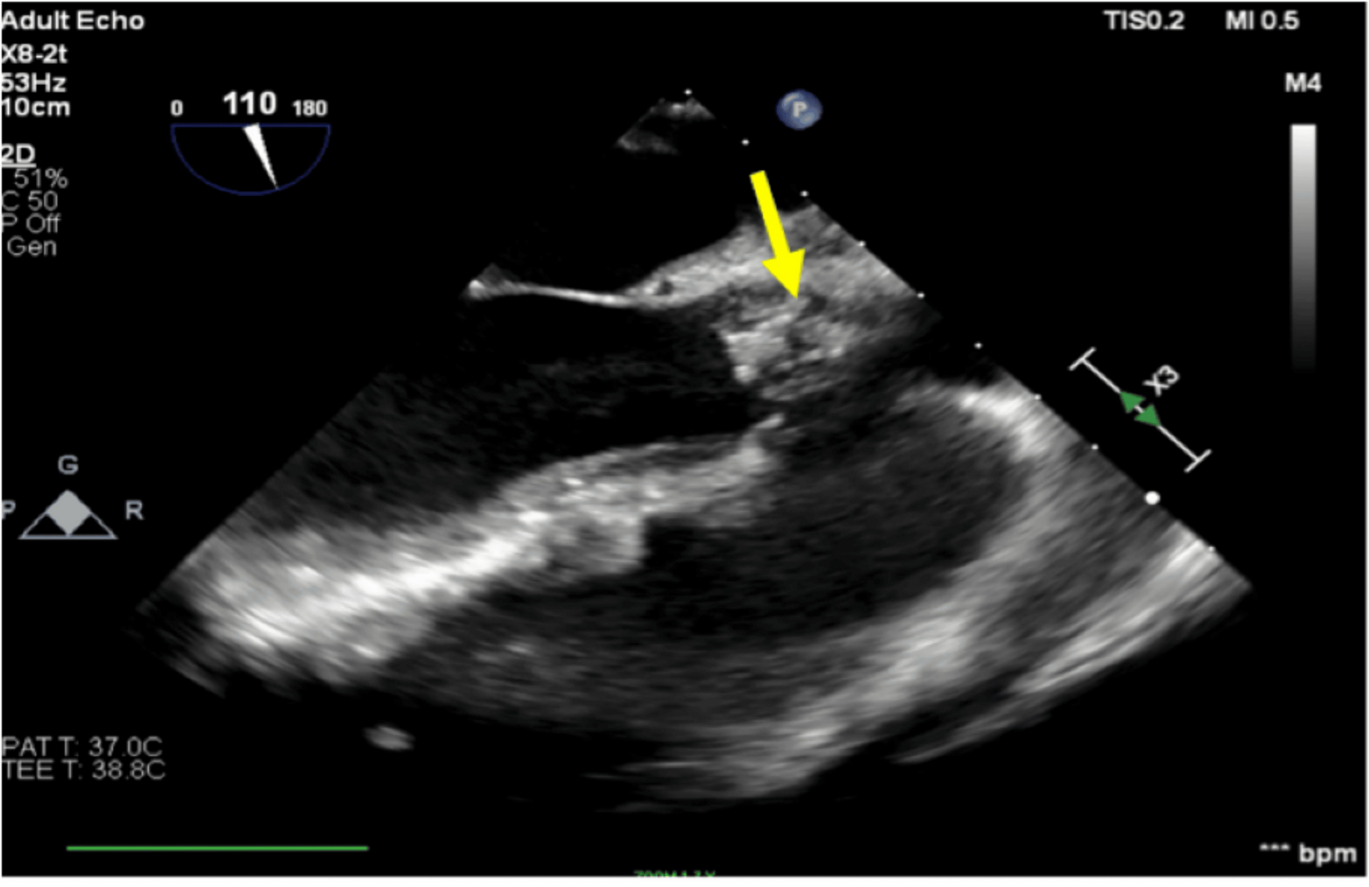
Transesophageal echocardiogram (TEE) findings TEE showing a long-axis view of the aortic valve and proximal aorta. The arrow shows a large conglomerate mass encasing the right and non-coronary cusps prolapsing towards the coronary ostia in systole.

**Figure 6:**
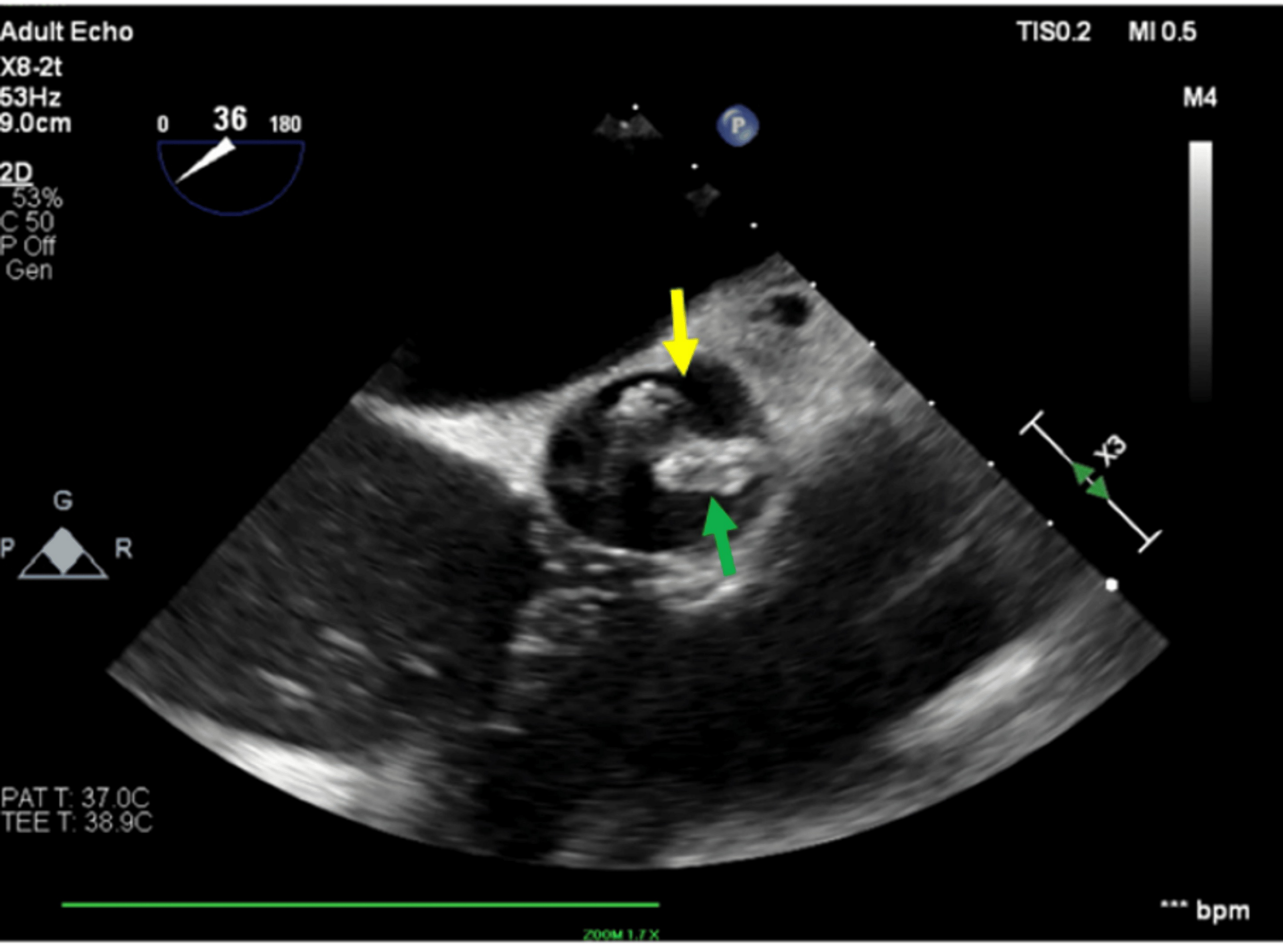
Transesophageal echocardiogram (TEE) short-axis view findings TEE showing a vegetation on the right coronary cusp (green arrow) and a smaller vegetation on the left coronary cusp. The yellow arrow shows the left main coronary artery.

**Figure 7:**
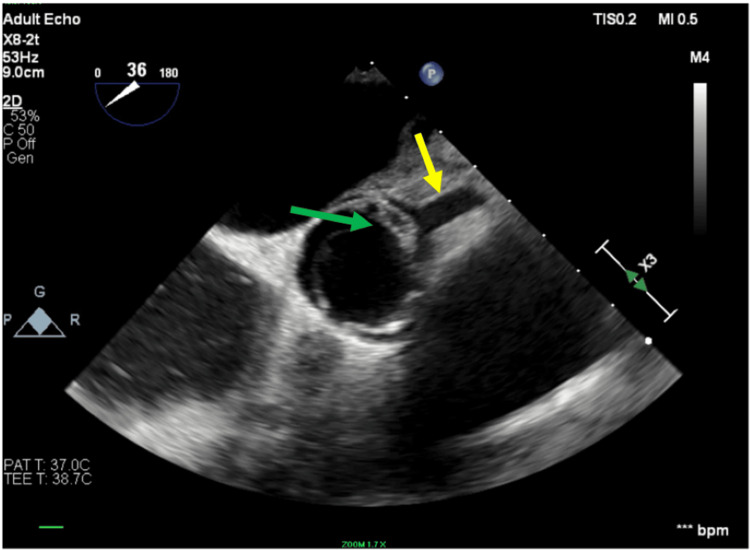
Transesophageal echocardiogram (TEE) short-axis view findings TEE showing the left main coronary artery (yellow arrow), and the green arrow shows the remnant of the vegetation at the ostium of the left main coronary artery.

Repeat TTE at our facility after transfer revealed extensive aortic valve endocarditis with large vegetation on the right coronary cusp (RCC) and the non-coronary cusp (NCC) (Figures 3, 4). Cardiothoracic surgery recommended a transesophageal echocardiogram (TEE) evaluation. An intraoperative TEE (Figure 5) during leg wound debridement confirmed complex aortic valve endocarditis with large vegetation on the left and NCC measuring approximately 2 X 1 cm and 1.2 X 0.8 cm. Smaller vegetations on the tricuspid and pulmonary valves, the largest vegetation prolapsing into the left main coronary artery (Figures 6, 7). A smaller vegetation was present on the RCC. These findings were consistent with widespread septicemia from the original wound and multivalvular seeding. Additionally, ejection fraction (EF) was 20% with akinesis of the anterolateral, lateral, and inferior walls, consistent with the left main occlusion and distal coronary embolization. On the day of wound debridement, the patient suffered cardiac arrest and passed.

## Discussion

We present a rare case of SCD as a serious complication in a young patient with MSSA endocarditis who presents with symptoms of chest pain, an abnormal ECG, and embolization down multiple coronary arteries from aortic valve vegetations. In the past 20 years, IVDU has been on the incline in the US, with the percentage of young (aged 15-34) patients hospitalized for IE increasing from 27.1% to 42% between 2000 and 2013 ​[9]. Among young people in the community diagnosed with endocarditis, the mortality rate is 16.3% in the hospital and 25.5% at six months ​[10]​. Cooper et al. reported that in 2021, 30 cases of SCD secondary to IE out of 6000 were reported. Postmortem analysis concluded that the cause of death in 43% of the 30 cases was septic emboli with myocardial infarction ​[8]. The true incidence of endocarditis associated with cardioembolic SCD may be underestimated because of concomitant comorbid conditions. 

The first report of a similar case was from 1988, with only seven total cases from outside of our patient. In 1988, Dowling and Buja reported the first case of SCD secondary to vegetation on the left coronary cusp of the aortic valve protruding into the left main coronary artery ​[11]​. Since this report, six other reports of SCD with coronary occlusion have been reported. In two cases reported by Millaire et al. (1996) and Brizzio et al. (2008), the patients survived with emergent surgical intervention ​[12,13]​. In the earlier case, Millaire et al. reported aortic vegetation that had grown 12 mm into the left main coronary artery, partially occluding it ​[12]​. In the more recent case, reported by Brizzio et al., the patient had a bicuspid aortic valve in addition to a fibrous mass that eventually grew Gram-positive cocci ​[13]​. In the remaining four cases, three cases of SCD were secondary to embolization of vegetation rather than protrusion, and the remaining patient had a prosthetic aortic valve. Our patient is differentiated from these cases as SCD was secondary to prolapsed vegetation occluding the left main coronary artery with a native aortic valve and no past medical history at admission. Goraya et al. describe a case of a torn bicuspid leaflet prolapsing into the left main coronary artery, causing chest pain that was urgently managed with valve replacement surgery and survived; there was no evidence of IE in this instance ​[14]​.

## Conclusions

Few studies discuss the prevalence of IE in young people who inject drugs (PWID), and fewer still propose mechanisms for cardiac complications. However, with the increase in PWID and cases of IE among this population, it is important to note this as a cause of SCD. The above case highlights the need to address IE urgently and documents a plausible mechanism of IE-associated SCD, including microembolization and coronary occlusion. Future research could focus on developing protocols for earlier imaging strategies, such as CT angiography in atypical presentations of IE, and systematic screening for coronary involvement in IE patients presenting with chest pain. This case is limited by its nature as a single-patient report and the absence of a postmortem autopsy, which prevents definitive determination of the exact mechanism of death.
